# Platelet-derived growth factor subunit-B mediating the effect of dickkopf-1 on acute myocardial infarction risk: a two-step Mendelian randomization study

**DOI:** 10.18632/aging.205413

**Published:** 2024-01-03

**Authors:** Jun-Shan Li, Peng-Fei Zheng, Jing-Jing Rong, Zhao-Fen Zheng, Zheng-Yu Liu, Chang-Lu Wang

**Affiliations:** 1 Cardiology Department, Hunan Provincial People’s Hospital Xingsha Branch (People’s Hospital of Changsha County), Changsha 410000, Hunan, China; 2 Cardiology Department, Hunan Provincial People’s Hospital, Changsha 410000, Hunan, China; 3 Clinical Research Center for Heart Failure in Hunan Province, Changsha 410000, Hunan, China; 4 Institute of Cardiovascular Epidemiology, Hunan Provincial People’s Hospital, Changsha 410000, Hunan, China

**Keywords:** DKK1, acute myocardial infarction, PDGF-B, two-sample Mendelian randomization, mediation analysis

## Abstract

Previous studies have indicated a potential connection between plasma levels of Dickkopf-1 (DKK1) and platelet-derived growth factor subunit-B (PDGF-B) with the development of atherosclerosis. However, the causal relationship between DKK1, PDGF-B, and the risk of acute myocardial infarction (AMI) is yet to be established. To address this research gap, we conducted Mendelian randomization (MR) and mediation analyses to investigate the potential mediating role of PDGF-B in the association between DKK1 and AMI risk. Summary statistics for DKK1 (n = 3,301) and PDGF-B (n = 21,758) were obtained from the GWAS meta-analyses conducted by Sun et al. and Folkersen et al., respectively. Data on AMI cases (n = 3,927) and controls (n = 333,272) were retrieved from the UK Biobank study. Our findings revealed that genetic predisposition to DKK1 (odds ratio [OR]: 1.00208; 95% confidence interval [CI]: 1.00056–1.00361; *P* = 0.0072) and PDGF-B (OR: 1.00358; 95% CI: 1.00136–1.00581; *P* = 0.0015) was associated with an increased risk of AMI. Additionally, genetic predisposition to DKK1 (OR: 1.38389; 95% CI: 1.07066–1.78875; *P* = 0.0131) was linked to higher PDGF-B levels. Furthermore, our MR mediation analysis revealed that PDGF-B partially mediated the association between DKK1 and AMI risk, with 55.8% of the effect of genetically predicted DKK1 being mediated through genetically predicted PDGF-B. These findings suggest that genetic predisposition to DKK1 is positively correlated with the risk of AMI, and that PDGF-B partially mediates this association. Therefore, DKK1 and PDGF-B may serve as promising targets for the prevention and treatment of AMI.

## INTRODUCTION

Acute myocardial infarction (AMI) has emerged as a leading cause of hospitalization and mortality worldwide, posing a significant threat to public health. Previous studies have suggested that atherosclerotic plaque rupture or vascular endothelial erosion, along with superimposed thrombosis formation, can lead to coronary artery occlusion, and subsequent necrosis of myocardial cells in the affected area due to ischemia and hypoxia [1, 2]. The pathogenesis of atherosclerosis has not been fully understood, but pathological studies have shown that it involves various processes such as vascular endothelial cell dysfunction, cytokine production, inflammatory cell infiltration, vascular smooth muscle cell proliferation and migration, and activation of macrophages and monocytes, all of which are closely associated with plaque stability [3]. Multiple studies suggest that vascular endothelial dysfunction represents the early stage of atherosclerosis and plays a crucial role in plaque regression and instability by inducing endothelial cell apoptosis and proinflammatory activation [4–6].

Dickkopf-1 (DKK1), a secretory glycoprotein, competitively binds to the LDL receptor-related protein 5 (LRP5) receptor on cell membranes, thereby blocking the Wnt signaling pathway [7]. Di et al. have demonstrated that DKK1 can promote endothelial dysfunction and apoptosis and regulate the expression of various inflammatory factors such as interleukin (IL)-6, IL-1β, tumor necrosis factor α (TNF-α), and monocyte chemoattractant protein-1 (MCP-1), all of which can contribute to plaque instability by increasing inflammation, collagen degradation, adhesion, and neovascularization [8] Additionally, an observational study involving 291 subjects has suggested that plasma DKK1 levels are higher in ST-segment elevation myocardial infarction (STEMI) patients than in non-ST-segment elevation acute coronary syndrome (NSE-ACS) patients. Furthermore, DKK1 plasma levels could serve as a prognostic predictor of the severity and stability of coronary atherosclerosis [9]. On the other hand, Li et al. have found that classic platelet-derived growth factors (PDGFs), such as PDGF subunit-A and -B (PDGF-A and -B), are overexpressed in various diseases, including fibrous diseases, cancer, and atherosclerosis [10]. Foam cell-derived PDGF-B, as a potent monocyte chemokine, has been shown to play a crucial role in the progression of human atherosclerotic plaque lesions [11, 12]. However, evidence on the direct correlation between DKK1, PDGF-B, and the risk of AMI is limited, and whether PDGF-B plays an intermediary role in the causal effect of DKK1 on the AMI risk remains unclear.

Observational research has inherent limitations such as confounding and reverse causality, necessitating the use of more effective methods to explore causality between features. Hence, Mendelian randomization (MR) is a widely used approach to infer causality by using genetic variations associated with the exposure of interest as instrumental variables (IVs) [13]. Since genetic variations are randomly assigned at conception, their relationship with outcomes is less likely to be influenced by environmental confounders. Furthermore, the MR mediation analysis employing a two-step approach has a potential in identifying the mediating role of certain factors in the causal relationship between exposures and outcomes, thereby reducing the common biases inherent in multivariable approaches [14, 15]. To the best of our knowledge, no previous study has established the causal relationship between DKK1, PDGF-B, and the risk of AMI using MR. Hence, this study was aimed to assess the causal effect of DKK1 and PDGF-B on the risk of AMI using the MR framework and identify the mediating effect of PDGF-B in the causal effect of DKK1 on the risk of AMI.

## RESULTS

### Selection and validation of SNPs

Several independent SNPs with pairwise linkage disequilibrium r2 < 0.001 were found to be significantly associated with DKK1 (rs1194673, rs6993770, and rs7080386) or PDGF-B (rs10761741, rs10876550, rs11594179, rs11639051, rs11770907, rs4541868, and rs68066031) at a genome-wide significance level of *p* < 5 × 10−8. All SNPs identified met the validity threshold (F > 10). Supplementary Table 1 provides further details on the characteristics of the SNPs and their associations with DKK1 and PDGF-B, as well as their correlation with the outcome of AMI.

### MR analysis

**Figure 1:**
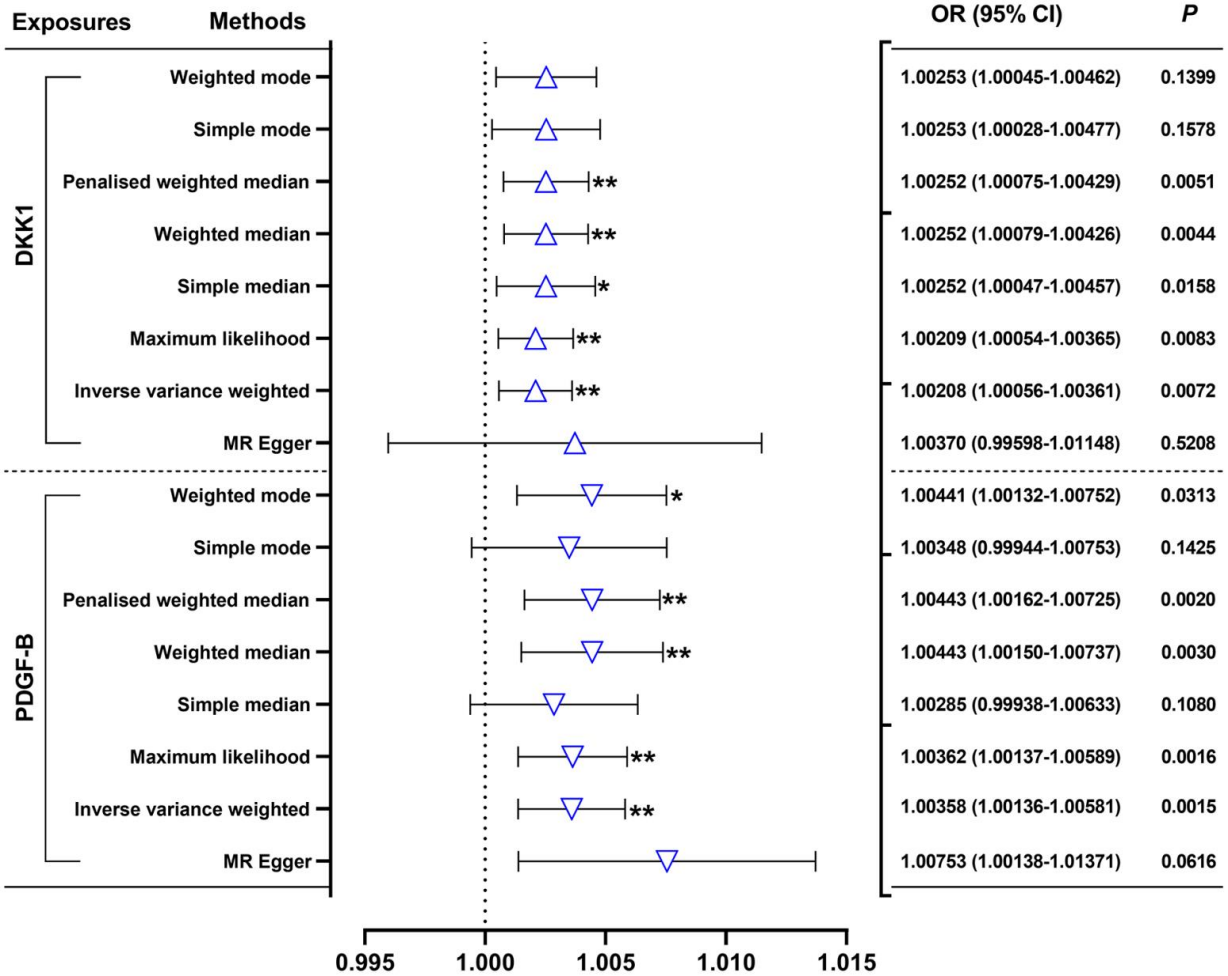
**Effects of genetically predicted DKK1 and PDGF-B on the AMI risk.** DKK1, dickkopf - 1; PDGF-B, platelet derived growth factor subunit-B; OR, odds ratio; CI, confidence interval. *P < 0.05, **P < 0.01.

**Figure 2:**
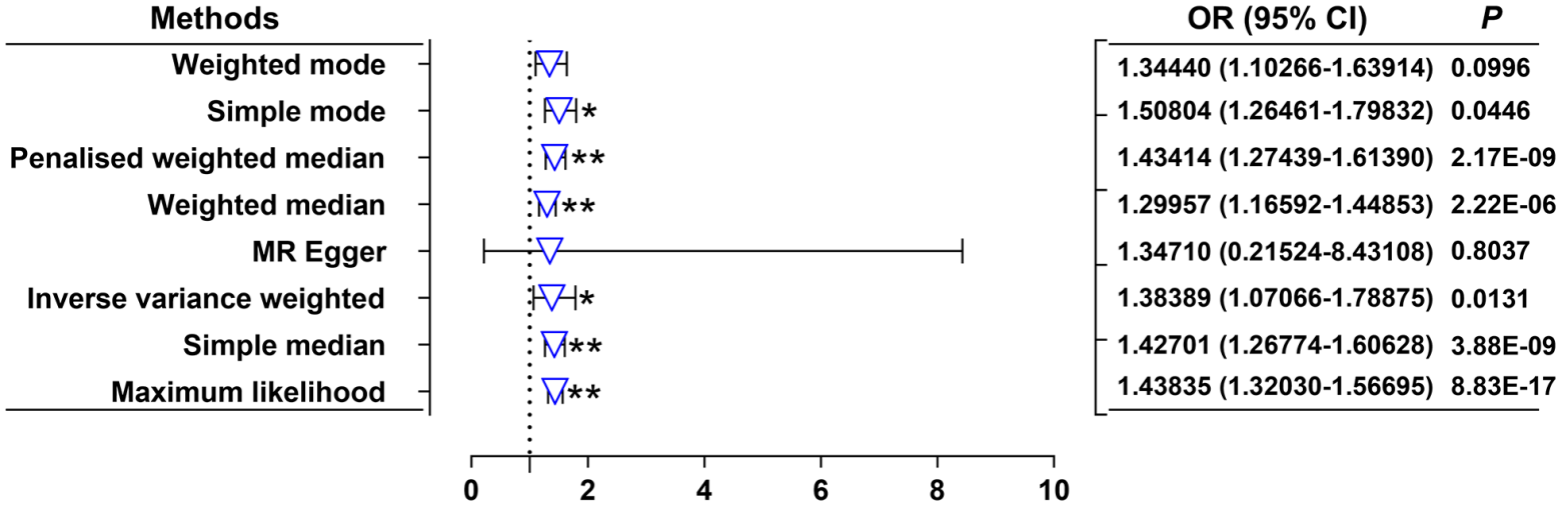
**Effects of genetically predicted DKK1 on the PDGF-B levels.** OR, odds ratio; CI, confidence interval. *P < 0.05, **P < 0.01.

Our random-effects IVW analysis revealed that both DKK1 (odds ratio [OR] = 1.00208; 95% confidence interval [CI]: 1.00056-1.00361; *P* = 0.0072) and PDGF-B (OR = 1.00358, 95% CI: 1.00136-1.00581; *P* = 0.0015) were significant risk factors for AMI (Figure 1 and Supplementary Table 2). Furthermore, we identified that DKK1 levels were a risk factor for PDGF-B levels (OR = 1.38389; 95% CI: 1.07066-1.78875; *P* = 0.0131) (Figure 2 and Supplementary Table 2). The scatter plot (Supplementary Figure 1) and forest plot (Supplementary Figure 2) showed a positive correlation between DKK1 and PDGF-B levels and the risk of AMI, while DKK1 levels were positively associated with PDGF-B levels. Sensitivity analysis using the leave-one-out method confirmed the causal effect between the levels of DKK1 and PDGF-B and the risk of AMI (Supplementary Figure 3A, 3B), as well as between the levels of DKK1 and PDGF-B (Supplementary Figure 3C). The TSMR analysis shows no heterogeneity (Supplementary Table 3) or horizontal pleiotropy (Supplementary Table 4) between DKK1 and PDGF-B levels and the risk of AMI, except for some heterogeneity in certain results (DKK1-PDGF-B). However, it did not invalidate the MR estimates as random-effect IVW in our study, which may balance the pooled heterogeneity. The mediation effect of PDGF-B in the effect of DKK1 on the risk of AMI was estimated to be 0.00116 (95% CI, 0.00001–0.00231; *P* = 0.009), with a mediated proportion of 55.8% (Table 1). Additionally, no causal effect was found between AMI and the levels of DKK1 and PDGF-B, or between PDGF-B and DKK1 levels (Supplementary Table 5).

**Table 1:** Mediation analysis of the mediation effect of DKK1 on the AMI risk via PDGF-B.

Total effect^*^	**Direct effect β1^†^**	**Direct effect β2^‡^**	**Mediation effect^§^**	
**Effect size (95% CI)**	**Effect size (95% CI)**	**Effect size (95% CI)**	**Effect size (95% CI)**	***P***
0.00208 (0.00056-0.00360)	0.32490 (0.06828-0.58152)	0.00358 (0.00136-0.00579)	0.00116 (0.00001-0.00231)	0.009

*Total effect: the effect of DKK1 on the AMI risk.

†Direct effect β1: the effect of the DKK1 on the PDGF-B.

‡Direct effect β2: the effect of the PDGF-B on the AMI risk.

§Mediation effect: the effect of DKK1 on the AMI risk acting through the PDGF-B.

DKK1, Dickkopf -1; PDGF-B, platelet derived growth factor subunit B; AMI, acute myocardial infarction.

## DISCUSSION

The present study revealed that a 10-standard deviation increase in DKK1 and PDGF-B levels was associated with a 2.08% and 3.58% increase in the risk of AMI, respectively. Additionally, we observed that a one standard deviation increases in DKK1 levels resulted in a 38.39% increase in PDGF-B levels. Furthermore, our mediation analysis indicated a mediation effect of PDGF-B of 0.00116 (95% CI, 0.00001–0.00231; *P* = 0.009) with a mediated proportion of 55.8% in the relationship between DKK1 and the risk of AMI. Notably, the TSMR analysis demonstrated no heterogeneity or horizontal pleiotropy between DKK1, PDGF-B levels, and the risk of AMI. Moreover, the consistency and robustness of our results, supported by the utilization of eight different methods, suggest an unconfounded and likely causal relationship between elevated levels of DKK1 and PDGF-B and the risk of AMI. Given the absence of horizontal pleiotropy but presence of heterogeneity in the TSMR analysis between DKK1 and PDGF-B, the random-effects IVW [16] and weighted median [17] methods were deemed more suitable for evaluating the causal relationship between them. Both methods unanimously supported a positive effect of DKK1 on PDGF-B levels. Overall, these findings suggest a causal effect between DKK1 levels and the risk of AMI, with approximately 55.8% of this effect mediated by PDGF-B. The robustness of our findings, along with the use of appropriate statistical methods, reinforces our conclusions and supports the potential of targeting DKK1 and PDGF-B as a promising approach to reduce the risk of AMI.

Our findings were consistent with numerous pathophysiology studies. Currently, DKK1 is considered as a biomarker of atherosclerosis. Its expression significantly increases in atherosclerotic lesions, leading to endothelial activation, inflammatory response, coronary atherosclerosis, and acute ischemic stroke [18–20]. As a secretory glycoprotein, DKK1 can inhibit the Wnt signaling pathway by competitively binding to the LRP5 receptor on the cell membrane, playing a non-lipid dependent role in vascular pathophysiology [7]. Various Wnt proteins participate in the process of atherosclerosis, with different subtypes exhibiting distinct functions. For instance, Wnt5a induces inflammation by activating the NF-κB transcriptional pathway in vascular endothelial cells [21]. In contrast, Wnt1 may act as an inhibitor of NF-κB activation, and compounds like geniposide and baicalin can enhance Wnt1 signaling by reducing DKK1 expression, further inhibiting the expression of downstream cytokines like interleukin-12 through the inhibition of NF-κB transcription factor activity, thus delaying the progression of atherosclerotic lesions [22]. Additionally, Ueland et al. highlighted DKK1 as a new mediator for platelet-mediated endothelial cell activation, underscoring its critical role in the pathological process of atherosclerosis through the inhibition of the Wnt/beta-catenin signaling pathway and the activation of the NF-kB pathway [18]. While these pieces of evidence emphasize the significance of DKK1 in atherosclerosis, further investigation is required to unravel its underlying mechanism.

Our findings are also supported by several observational studies. For instance, Ueland et al. conducted a clinical study that demonstrated significantly increased serum DKK1 levels in patients with angina pectoris compared to healthy subjects. Furthermore, patients with unstable angina pectoris exhibited higher serum DKK1 levels than those with stable angina pectoris and healthy individuals [18]. In another observational study involving 291 subjects, higher plasma DKK-1 levels were observed in STEMI patients compared to NSE-ACS patients. The study also suggested that DKK1 plasma levels could serve as a prognostic predictor for the severity and stability of coronary atherosclerosis [9]. Goliach et al. conducted a case-control study of 100 young patients with myocardial infarction and 100 healthy controls, revealing that elevated DKK1 expression levels significantly increased the risk of early-onset myocardial infarction [23]. Additionally, a clinical study by Zhu et al. included 3178 patients with ischemic stroke (IS), finding a significant association between increased DKK1 levels and adverse prognoses of all-cause mortality and severe disability one year after IS [24]. These evidences demonstrated the pivotal role of DKK1 in atherosclerotic diseases. However, observational studies are inadequate for establishing causality due to susceptibility to confounding factors and reverse causal bias. Therefore, the direct causal relationship between elevated DKK1 levels and the risk of AMI remains uncertain. In our study, we observed a significant genetic association between plasma DKK1 levels and AMI, providing a direct causal estimation of the impact of DKK1 levels on AMI risk in the European population. Furthermore, we quantified the mediating effects of genetically-predicted PDGF-B among the effects of DKK1 on AMI risk.

Previous research has emphasized the crucial role of PDGF in various pathological conditions, including fibrosis, neurological disorders, cancer, and atherosclerosis. Dysfunctional PDGF signaling can lead to various cellular responses, such as cell proliferation, migration, and survival, all of which contribute to the pathogenesis of atherosclerosis [25]. Cell-to-cell interaction between monocytes and vascular endothelial cells results in the synthesis of PDGF in both cell types. This synthesized PDGF promotes the migration and proliferation of vascular smooth muscle cells, thereby playing a pivotal role in the pathogenesis of atherosclerosis [26]. PDGF comprises four subtypes: PDGF-A, PDGF-B, PDGF-C, and PDGF-D, all of which can participate in atherosclerosis by enhancing MMP activity and influencing monocyte migration [27]. Candido et al. found that the expression levels of PDGF-B increased significantly in diabetes-associated atherosclerotic lesions [28]. Coombes et al. observed that PDGF-B may act as a key molecule in the development of atherosclerosis caused by chlamydia pneumoniae infection [29]., by mediating the proliferation of smooth muscle cells and intimal thickening. Wang et al. reported a significant upregulation of PDGF-B expression in the ischemic myocardium of rats [30]. However, Zhao et al. found that the expression of PDGF-A and PDGF-D significantly increased in the infarcted myocardium, whereas the expression of PDGF-B and PDGF-C decreased significantly in the rat model of myocardial infarction [31]. Although the relationship between PDGF-B and AMI is controversial, our TSMR analysis, based on eight different algorithms, demonstrated for the first time that there is a causal relationship between evaluated PDGF-B levels and the risk of AMI. Our analysis revealed that a 10-standard deviation increase in PDGF-B levels was associated with a 3.58% increase in the risk of AMI. These results suggest that PDGF-B is a promising target for reducing the risk of AMI.

Currently, there has been no reported direct association between DKK1 and PDGF-B levels, and the influence of DKK1 on the expression of PDGF-B has also remained unknown. Previous studies highlighted the crucial role of crosstalk between Wnt/β-catenin and NF-κB signaling pathways in the inflammatory response [32]. Various studies have demonstrated the inhibiting effects of the Wnt/β-catenin pathway on NF-κB activity and downstream genes in various cells and tissues [33–35]. Besides, a compelling study suggests that DKK1, as an inhibitor of the canonical Wnt/β-catenin pathway, may enhance the activity of the NF-κB inflammatory pathway. Inhibition of DKK1 can obviously reduce the expression of IL-8 and MCP-1 in platelet-mediated endothelial cells, thereby improving the stability of atherosclerotic plaques [18]. NF-κB signaling pathway plays a critical role in the atherosclerosis process induced by lipid metabolism disorders [36], and NF-κB maybe a key transcription factor responsible for the mediation of hypercholesterolemia-induced PDGF-B expression [37]. These findings suggest that the DKK1/Wnt/NF-κB/PDGF-B pathway may be sequentially interconnected to form a complete pathway that plays a pivotal role in atherosclerotic diseases. In our study, we utilized TSMR analysis to evaluate the association between serum DKK1 and PDGF-B levels, and results of six different methods, including random-effects IVW, maximum likelihood, simple median, weighted median, penalized weighted median, and simple mode, all support a causal relationship between serum DKK1 levels and PDGF-B levels. Based on these results, it could be inferred that DKK1 can exert its critical biological function by regulating the expression of PDGF-B.

Our study had several limitations that should be considered. Firstly, the effect estimates of SNPs on DKK1 and PDGF-B and their association with AMI were derived from individuals of European ancestry. Therefore, the generalizability of our findings to other ethnic groups may be limited. Additional research would be needed to determine the causal relationship between DKK1 and PDGF-B levels and the risk of AMI in different populations. Secondly, since our study found that PDGF-B mediated the effect of DKK1 on AMI risk, the regulatory mechanism of DKK1/Wnt/NF-κB/PDGF-B signaling pathway in atherosclerotic diseases necessitates additional *in vitro* and *in vivo* research for further investigation in future.

In conclusion, our findings demonstrated that the genetic susceptibility of DKK1 was positively correlated with the risk of AMI. Besides, this association was partially mediated by PDGF-B. These results highlighted the potential of DKK1 and PDGF-B as novel targets for the prevention and treatment of AMI.

## MATERIALS AND METHODS

### Data sources

In this study, we utilized data from two GWAS meta-analyses and the United Kingdom Biobank. We incorporated genetic associations related to the DKK1 protein from the GWAS meta-analysis conducted by Sun et al. [38], which quantified 3,622 plasma proteins, including DKK1, using 3,301 healthy subjects extracted from the INTERVAL study. The summary statistics for PDGF-B protein were obtained from the GWAS meta-analysis reported by Folkersen et al. [39] which involved a meta-analysis of 90 proteins based on 21,758 individuals from 13 cohorts. The outcome data for AMI was extracted from the United Kingdom Biobank, consisting of 3,927 cases and 333,272 controls, and supported by the Neale Lab Consortium (http://www.nealelab.is/). All GWAS data for DKK1 (prot-a-821), PDGF-B (ebi-a-GCST90012016), and AMI (ukb-a-533) are publicly available at the MRC IEU OpenGWAS data infrastructure [40] (https://gwas.mrcieu.ac.uk). However, the GWAS datasets used in this study were based on European populations. Ethical approval was not required for this secondary analysis of public data.

### Study design and selection of IVs

**Figure 3:**
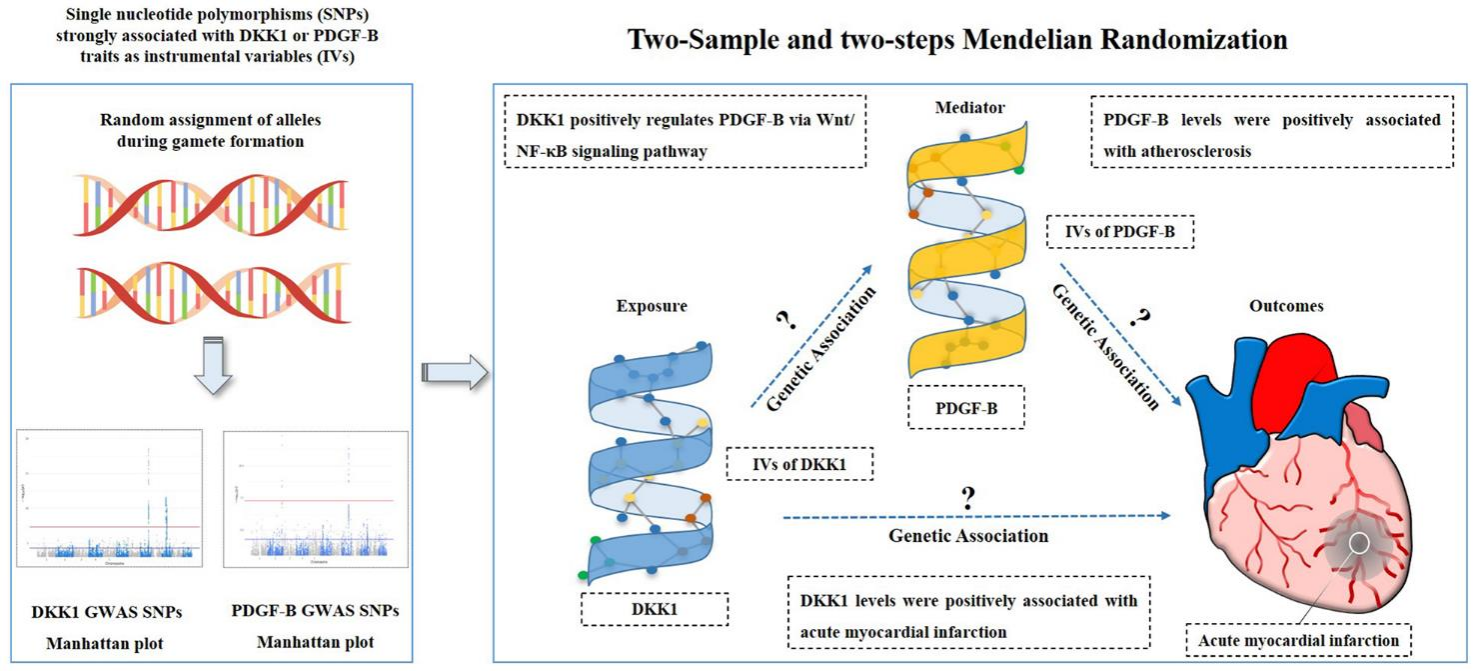
**Study hypothesis and flow chart.** DKK1, Dickkopf - 1; PDGF-B, platelet derived growth factor subunit-B.

The study hypothesis and flow chart are depicted in Figure 3. We used a two-sample MR (TSMR) analysis [41] to evaluate the causal relationship between the exposures and the outcome, and then identified the effects of potential mediation using two-step MR [15]. In step one, we assessed the causal effect of DKK1 on PDGF-B levels and the risk of AMI. Subsequently, we evaluated the causal effect of PDGF-B on the risk of AMI in step two. We selected single nucleotide polymorphisms (SNPs) significantly associated with DKK1 and PDGF-B exposures from the corresponding GWAS as IVs using a genome-wide significance threshold of *P* < 5 × 10^−8^. To ensure the independence of each SNP from one another, we considered a pairwise linkage disequilibrium (LD) r2 < 0.001 with a distance of kb = 10000 between them. To avoid weak instrument bias due to the small number of IVs in DKK1, F-statistic was calculated to validate the strength of each SNP based on the R2 (i.e., the proportion of phenotypic variance explained by each SNP) [13]. An instrument is weak if the F-statistic in the SNP to exposure regression is less than 10 [42]. All IVs were extracted from the GWAS using the TwoSampleMR package in R [43].

### MR analysis

Due to limited availability of comprehensive data within a single cohort, we performed a TSMR approach to analyze data from two distinct samples, one for the exposure of interest and the other for the outcome [44]. We employed eight MR methods, including inverse-variance weighted (IVW), MR-Egger, penalized weighted median, simple mode, simple median, maximum likelihood, weighted mode, and weighted median, to compute follow-up sensitivity [45, 46]. The IVW test, a random-effects model, was used to estimate the primary causal effect values when there was no horizontal pleiotropy, providing unbiased estimates [13]. The remaining seven MR methods were supplementary approaches for MR analysis and were more resilient to individual genetics with strong outlier causal estimates. These methods produced consistent estimates of causal effects when the effective IVs exceeded 50% [43]. By leveraging the strengths of each MR method, these eight approaches complemented one another and provided reliable causal effects for our research when the direction of β values was consistent.

For the preliminary analysis, we assessed the causal effect of DKK1 on PDGF-B levels, as well as the effect of DKK1 and PDGF-B on the risk of AMI using the random-effects IVW method. We performed a leave-one-out sensitivity analysis to verify the reliability and stability of the causal effect estimates. We employed the MR-Egger intercept test to evaluate the presence of horizontal pleiotropy and used Cochran’s Q test to test the heterogeneity between genetic variants. The intermediary effect value of PDGF-B on the DKK1-AMI risk was determined by multiplying the effect value (β1) of DKK1 on PDGF-B levels and the effect value (β2) of PDGF-B on the AMI risk. We excluded potential confounding SNPs using PhenoScanner [47] and performed these analyses using the “TwoSampleMR” R package [43].

## Supplementary Material

Supplementary Figures

Supplementary Table 1

Supplementary Table 2

Supplementary Tables 3 and 4

Supplementary Table 5
