# Supplementary material for: Platelet-derived growth factor subunit-B mediating the effect of dickkopf-1 on acute myocardial infarction risk: a two-step Mendelian randomization study
Source: Aging (Albany NY). 2024 Jan 3;16(1):701–13. doi: 10.18632/aging.205413 (PMC10817415; doi:10.18632/aging.205413)
Supplement: Supplementary Figures [file aging-16-205413-s001.pdf]

SUPPLEMENTARY FIGURES

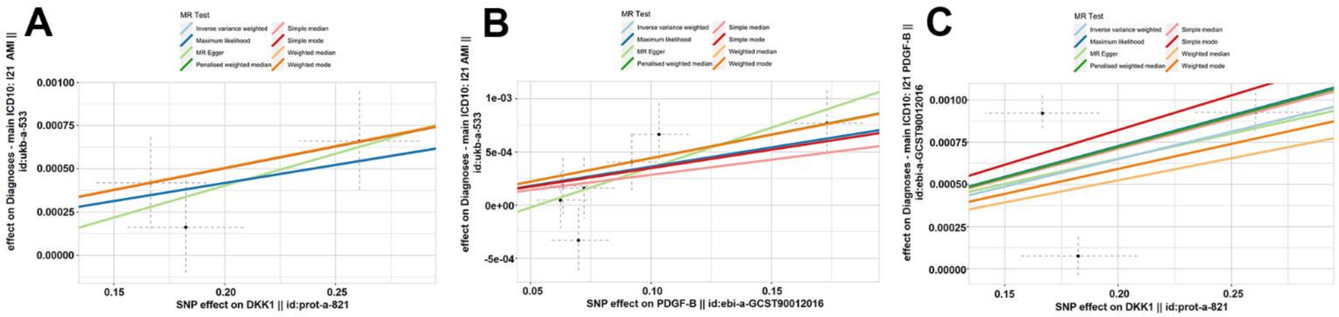

Supplementary Figure 1. Scatter plots of the estimated SNP effects on exposures (x-axis) plotted against the estimated SNPs effects on the outcome (y-axis). (**A**) DKK1-AMI; (**B**) PDGF-B-AMI; (**C**) DKK1PDGF-B. The slope of the line corresponds to a causal estimate using a different method. DKK1, dickkopf-1; PDGF-B, platelet derived growth factor subunit-B; AMI, acute myocardial infarction; SNP, single nucleotide polymorphism.

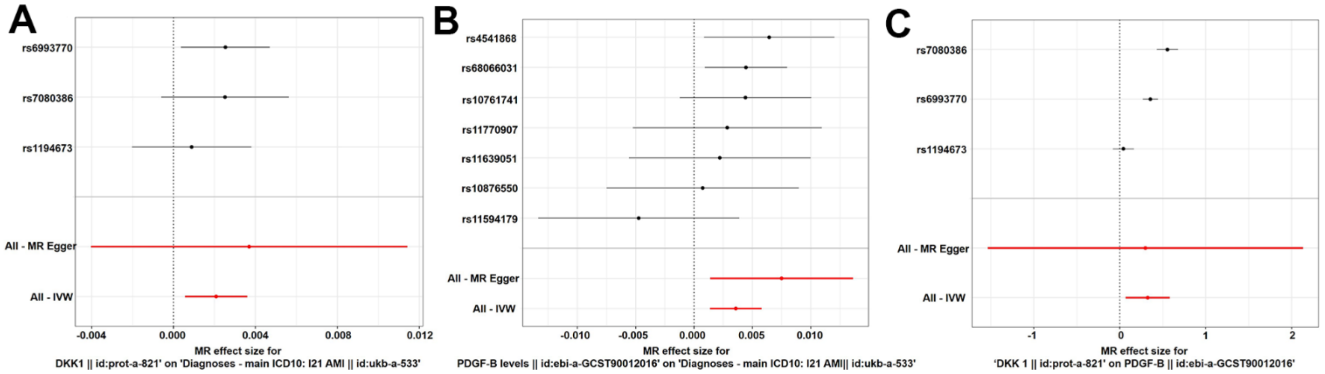

Supplementary Figure 2. Results of the single and multi SNP analyses for the SNP effect of exposures on AMI. (**A**) DKK1-AMI; (**B**) PDGF-B-AMI; (**C**) DKK1-PDGF-B. The forest map, where each black dot represented a single SNP as instrumental variable and the red dot showed the use of IVW results for all SNPs. DKK1, Dickkopf-1; PDGF-B, platelet derived growth factor subunit-B; AMI, acute myocardial infarction; SNP, single nucleotide polymorphism.

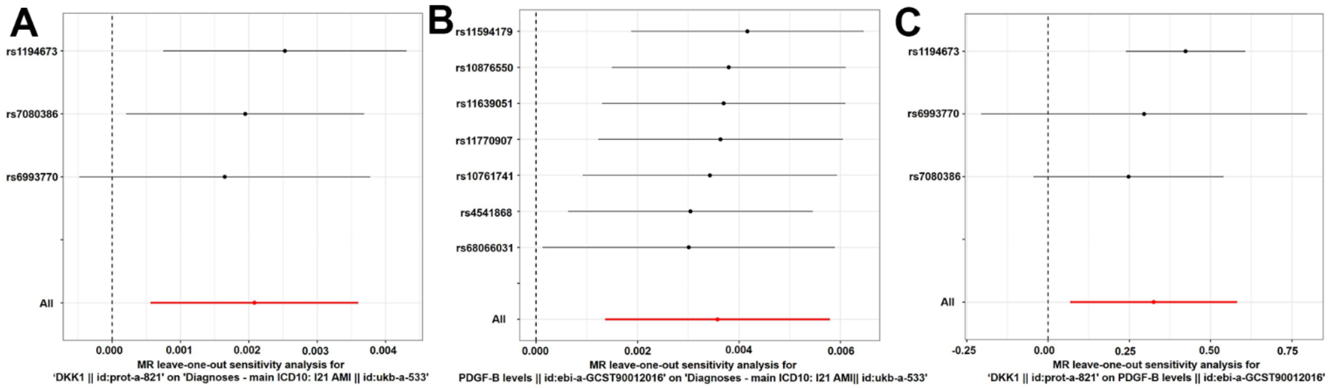

Supplementary Figure 3. Sensitivity analyses using the leave-one-out approach on the association of exposures on outcome. (**A**) DKK1-AMI; (**B**) PDGF-B-AMI; (**C**) DKK1-PDGF-B. Each black dot represents an IVW method for estimating causal the effect of the exposures on the AMI does not exclude a case where a particular SNP caused a significant change in the overall results. DKK1, dickkopf-1; PDGF-B, platelet derived growth factor subunit-B; AMI, acute myocardial infarction; SNP, single nucleotide polymorphism.
