# Supplementary material for: Platelet-derived growth factor subunit-B mediating the effect of dickkopf-1 on acute myocardial infarction risk: a two-step Mendelian randomization study
Source: Aging (Albany NY). 2024 Jan 3;16(1):701–13. doi: 10.18632/aging.205413 (PMC10817415; doi:10.18632/aging.205413)
Supplement: Supplementary Tables 3 and 4 [file aging-16-205413-s004.pdf]

Supplementary Table 3. The results of heterogeneity analysis.

Exposures	Outcome	Method	Q	P-value
DKK1	AMI	MR Egger	0.711419129041146	0.398973097650447
DKK1	AMI	Inverse variance weighted	0.885058635326956	0.642409506746299
DKK1	PDGF-B	MR Egger	34.1135889192556	5.19869493201528E-09
DKK1	PDGF-B	Inverse variance weighted	34.1430312544289	3.85420637468344E-08
PDGF-B	AMI	MR Egger	3.70285392227648	0.592939312824997
PDGF-B	AMI	Inverse variance weighted	5.51597590261115	0.479527906565962

DKK1, Dickkopf-1; PDGF-B, platelet derived growth factor subunit-B; AMI, acute myocardial infarction.

Supplementary Table 4. The results of priority analysis.

Exposures	Outcome	Egger intercept	Standard error	P-value
DKK1	AMI	-0.000335534213356646	0.000805216265689418	0.748649684830127
DKK1	PDGF-B	0.00560576274859444	0.190814903890872	0.98130275373835
PDGF-B	AMI	-0.000396813594302379	0.000294695178656892	0.235950262013081

DKK1, dickkopf-1; PDGF-B, platelet derived growth factor subunit-B; AMI, acute myocardial infarction.
